# Supplementary material for: Effects of the killer immunoglobulin–like receptor (KIR) polymorphisms on HIV acquisition: A meta-analysis
Source: PLoS One. 2019 Dec 2;14(12):e0225151. doi: 10.1371/journal.pone.0225151 (PMC6886768; doi:10.1371/journal.pone.0225151)
Supplement: S1 List — (DOCX) [file pone.0225151.s001.docx]

**Supplementary list 1**

**1) Abstract screening (n=34)**

**1.1 HIV progression, HIV outcome and HIV-infected vs healthy control studies were excluded (N=26).**

1. Inhibitory natural killer cell receptor KIR3DL1 with its ligand Bw4 constraints HIV-1 disease among South Indians. **Maruthamuthu S, AIDS. 2018**
2. [Killer-cell immunoglobulin-like receptors associate with **HIV**-1 infection in a narrow-source Han Chinese cohort.](https://www.ncbi.nlm.nih.gov/pubmed/29664957) **Wang L, PLoS One. 2018**
3. [Impact of HLA Allele-KIR Pairs on Disease Outcome in HIV-Infected Thai Population.](https://www.ncbi.nlm.nih.gov/pubmed/29528943)

**Mori M.J Acquir Immune Defic Syndr. 2018**

1. [Killer cell immunoglobulin-like receptor 3DL1 variation modifies HLA-B*57 protection against HIV-1.](https://www.ncbi.nlm.nih.gov/pubmed/29461980) **Martin MP, J Clin Invest. 2018**

5. [Distribution of killer immunoglobulin-like receptor genes in HIV infected long-term non-progressors from Mumbai, India.](https://www.ncbi.nlm.nih.gov/pubmed/29327701) **Chavan VR, indian J Dermatol Venereol Leprol. 2018**

6. [Activating Killer Immunoglobulin Receptors and HLA-C: a successful combination providing **HIV-1** control.](https://www.ncbi.nlm.nih.gov/pubmed/28211903) **Malnati MS, Sci Rep. 2017**

7. [KIR Gene Content Diversity in a Zimbabwean Population: Does KIR2DL2 Have a Role in Protection Against Human Immunodeficiency Virus Infection?](https://www.ncbi.nlm.nih.gov/pubmed/27930093) **Mhandire K. OMICS. 2016 Dec;20(12):727-735.**

8. [Association between KIR genotypes and HLA-B alleles on viral load in Southern Brazilian individuals infected by HIV-1 subtypes B and C.](https://www.ncbi.nlm.nih.gov/pubmed/27346697) **Fernandes-Cardoso J, Hum Immunol. 2016**

9. [Killer cell immunoglobulin-like receptor and human leukocyte antigen gene profiles in a cohort of HIV-infected Mexican Mestizos.](https://www.ncbi.nlm.nih.gov/pubmed/27240860) **Garrido-Rodríguez D, Immunogenetics. 2016**

10. [Killer Cell Immunoglobulin-Like Receptor Alleles Alter HIV Disease in Children.](https://www.ncbi.nlm.nih.gov/pubmed/26983081)

**Singh KK, PLoS One. 2016**

11. [Association of KIR3DL1/S1 and HLA-Bw4 with CD4 T cell counts in HIV-infected Mexican mestizos.](https://www.ncbi.nlm.nih.gov/pubmed/26033692) **Hernández-Ramírez D, Immunogenetics. 2015**

12.[The HLA-C*04: 01/KIR2DS4 gene combination and human leukocyte antigen alleles with high population frequency drive rate of HIV disease progression.](https://www.ncbi.nlm.nih.gov/pubmed/25715101) **Olvera A, AIDS. 2015**

13. [KIR2DS4 promotes **HIV**-1 pathogenesis: new evidence from analyses of immunogenetic data and natural killer cell function.](https://www.ncbi.nlm.nih.gov/pubmed/24901871) **Merino AM, PLoS One. 2014**

14. [KIR3DS1/L1 and HLA-Bw4-80I are associated with HIV disease progression among HIV typical progressors and long-term nonprogressors.](https://www.ncbi.nlm.nih.gov/pubmed/24059286) **Jiang Y. BMC Infect Dis. 2013**

15. [Associations of HLA class I antigen specificities and haplotypes with disease progression in HIV-1-infected Hans in Northern China.](https://www.ncbi.nlm.nih.gov/pubmed/24012585) **Zhang H, Hum Immunol.2013**

16. [Variation in both IL28B and KIR2DS3 genes influence pegylated interferon and ribavirin hepatitis C treatment outcome in HIV-1 co-infection.](https://www.ncbi.nlm.nih.gov/pubmed/23826153) **Keane C, PLoS One. 2013**

17. [Genetic variations in loci relevant to natural killer cell function are affected by ethnicity but are generally not correlated with susceptibility to HIV-1.](https://www.ncbi.nlm.nih.gov/pubmed/22296096) **Aghafar MZ, Tissue Antigens. 2012**

18. [Copy number variation of KIR genes influences HIV-1 control.](https://www.ncbi.nlm.nih.gov/pubmed/22140359) **Pelak K, PLoS Biol. 2011**

19. [Low CD4+ T cell counts among African HIV-1 infected subjects with group B KIR haplotypes in the absence of specific inhibitory KIR ligands.](https://www.ncbi.nlm.nih.gov/pubmed/21347267) **Jennes W, PLoS One. 2011**

20. [Influence of HLA class I and HLA-KIR compound genotypes on HIV-2 infection and markers of disease progression in a Manjako community in West Africa.](https://www.ncbi.nlm.nih.gov/pubmed/20519398) **Yindom LM. J Virol. 2010**

21. [Innate partnership of HLA-B and KIR3DL1 subtypes against HIV-1.](https://www.ncbi.nlm.nih.gov/pubmed/17496894) **Martin MP, Nat Genet. 2007**

22. [KIR/HLA pleiotropism: protection against both HIV and opportunistic infections.](https://www.ncbi.nlm.nih.gov/pubmed/16933987)

**Qi Y, PLoS Pathog. 2006**

23. [Killer immunoglobulin-like receptors and HLA act both independently and synergistically to modify HIV disease progression.](https://www.ncbi.nlm.nih.gov/pubmed/16121209) **Gaudieri S, Genes Immun. 2005**

24. [Interaction between KIR3DL1 and HLA-B*57 supertype alleles influences the progression of HIV-1 infection in a Zambian population.](https://www.ncbi.nlm.nih.gov/pubmed/15784466)**López-Vázquez A**, **Hum Immunol. 2005**

25. [Epistatic interaction between KIR3DS1 and HLA-B delays the progression to AIDS.](https://www.ncbi.nlm.nih.gov/pubmed/12134147) **Martin MP, Nat Genet. 2002**

26. [Control of HIV-1 viremia and protection from AIDS are associated with HLA-Bw4 homozygosity.](https://www.ncbi.nlm.nih.gov/pubmed/11309482)**Flores-Villanueva PO, Proc Natl Acad Sci U S A. 2001**

**1.2) Study groups involved in other disease (HESN with hemophilia A, N=1)**

1. [HLA class I and KIR genes do not protect against HIV type 1 infection in highly exposed uninfected individuals with hemophilia A.](https://www.ncbi.nlm.nih.gov/pubmed/24719475) **Vince N, J Infect Dis. 2014**

**1.3) Mother to child/paediatric studies were excluded (N= 7).**

1. [Association of maternal KIR gene content polymorphisms with reduction in perinatal transmission of HIV-1.](https://www.ncbi.nlm.nih.gov/pubmed/29360870)**Omosun YO, PLoS One. 2018**
2. [Killer Cell Immunoglobulin-Like Receptor Alleles Alter HIV Disease in Children.](https://www.ncbi.nlm.nih.gov/pubmed/26983081) **Singh KK, PLoS One. 2016**
3. [Diversity in KIR gene repertoire in HIV-1 exposed infected and uninfected infants: A study from India.](https://www.ncbi.nlm.nih.gov/pubmed/26255774) **Chavan VR, Ahir J Med Virol. 2016**
4. [KIR2DS4 allelic variants: Differential effects on in utero and intrapartum HIV-1 mother-to-child transmission.](https://www.ncbi.nlm.nih.gov/pubmed/24239756) **Hong HA, Clin Immunol. 2013**
5. [Differential association of gene content polymorphisms of killer cell immunoglobulin-like receptors with placental malaria in HIV- and HIV+ mothers.](https://www.ncbi.nlm.nih.gov/pubmed/22715396) **Omosun YO, PLoS One. 2012**
6. [KIR-HLA and maternal-infant HIV-1 transmission in sub-Saharan Africa.](https://www.ncbi.nlm.nih.gov/pubmed/21346814) **Paximadis M, PLoS One. 2011.**
7. [Mother-to-child transmission of HIV-1: strong association with certain maternal HLA-B alleles independent of viral load implicates innate immune mechanisms.](https://www.ncbi.nlm.nih.gov/pubmed/15167284) **Winchester R, J Acquir Immune Defic Syndr. 2004**

**2) Full-text articles assessed for eligibility**

**2.1 Imcomplete/ absent data/ not available (N=4)**

1. [Selection of an HLA-C*03:04-Restricted HIV-1 p24 Gag Sequence Variant Is Associated with Viral Escape from KIR2DL3+ Natural Killer Cells: Data from an Observational Cohort in South Africa.](https://www.ncbi.nlm.nih.gov/pubmed/26575988) **Hölzemer A, PLoS Med. 2015**

2. [In vivo evidence insufficient to conclude that "KIR/HLA incompatibility between sexual partners confers protection against HIV-1 transmission".](https://www.ncbi.nlm.nih.gov/pubmed/24030258) **Behrendt CE, Zaia JA. Blood. 2013.**

3. [Inhibitory KIR/HLA incompatibility between sexual partners confers protection against HIV-1 transmission.](https://www.ncbi.nlm.nih.gov/pubmed/23243280) **Jennes W, Blood. 2013**

4. [Distribution of natural killer cell receptors in HIV infected individuals.](https://www.ncbi.nlm.nih.gov/pubmed/17908466) **Jiang YJ, Chin Med J (Engl). 2007**
