# Supplementary material for: Effects of the killer immunoglobulin–like receptor (KIR) polymorphisms on HIV acquisition: A meta-analysis
Source: PLoS One. 2019 Dec 2;14(12):e0225151. doi: 10.1371/journal.pone.0225151 (PMC6886768; doi:10.1371/journal.pone.0225151)
Supplement: S1 Table — (DOCX) [file pone.0225151.s002.docx]

**Supplementary Table**

**S1 Table** Characteristics of the studies in the *KIR* gene content polymorphisms and their associations with HIV acquisition

| **First author** | **Year** | **Country** | **Ethnic** | **N** | **HIVI** | **HESN** | **KIR** | **CB** | ***KIR* gene content polymorphisms** |
| --- | --- | --- | --- | --- | --- | --- | --- | --- | --- |
|  |  |  | **Group** |  |  |  | **Genotyping** |  |  |
| **Merino** | **2011** | Zambia | African | 566 | 240 | 326 | PCR-SSP | 8 | *2DL1-3, 2DL5A,2DL5B, 2DS1-3, 2DS4F, 2DS4D,2DS5,3DL1, 3DS1* |
| **Koehler** | **2013** | Tanzania | African | 271 | 174 | 97 | PCR-SSP | 8 | *2DL2-3, 3DL1, 3DS1* |
| **Chavan** | **2014** | India | Asian | 94 | 47 | 47 | PCR-SSP | 7 | *2DL1-3, 2DL5A, 2DL5B, 2DS1-3, 2DS4F, 2DS4D, 2DS5, 3DL1, 3DS1* |
| **Mori** | **2015** | Thailand | Asian | 313 | 209 | 104 | NA | 6 | *2DL1-3, 2DS1-2, 3DL1, 3DS1* |
| **Zwolinska** | **2016** | Poland | Caucasian | 577 | 459 | 118 | PCR-SSP | 6 | *2DL1-3, 2DL5, 2DS1-3, 2DS4F, 2DS4D, 2DS5, 3DL1, 3DS1* |
| **Naranbhai** | **2016** | South Africa | African | 309 | 154 | 155 | PCR-SSO | 8 | *2DL1-3, 2DL5, 2DS1-3, 2DS5,3DL1, 3DS1* |
| **Jackson** | **2017** | Canada | Caucasian | 220 | 123 | 97 | PCR-SSP | 8 | *2DL1-3, 2DL5, 2DL5A, 2DL5B,2DS1-3, 2DS4F, 2DS4D, 2DS5, 3DL1, 3DS1* |
| **Rallon** | **2017** | Spain | Caucasian | 90 | 61 | 29 | PCR-SSO | 5 | *2DL1-3, 2DL5, 2DS1-3, 2DS4F, 2DS4D, 2DS5, 3DL1, 3DS1* |

N; number of samples, HIV: Human Immunodeficiency Virus; HIVI: HIV-Infected; HESN: *HIV-exposed seronegative;*  NA; not available, PCR-SSP: Polymerase Chain Reaction –Sequence Specific

Primer; PCR-SSO: Polymerase Chain Reaction- Sequence Specific Oligonucleotides; CB: Clark- Baudouin.
