# Supplementary material for: Effects of the killer immunoglobulin–like receptor (KIR) polymorphisms on HIV acquisition: A meta-analysis
Source: PLoS One. 2019 Dec 2;14(12):e0225151. doi: 10.1371/journal.pone.0225151 (PMC6886768; doi:10.1371/journal.pone.0225151)
Supplement: S2 Table — (DOCX) [file pone.0225151.s003.docx]

**Supplementary Table**

**S2 Table** Characteristics of the studies in the *3DL1/S1* genotype polymorphisms and their associations with HIV acquisition

| **First author** | **Year** | **Country** | **Ethnic Group** | **N** | **HIVI** | **HESN** | **KIR** | **CB** | **HIVI** | | |  | **HESN** | | |
| --- | --- | --- | --- | --- | --- | --- | --- | --- | --- | --- | --- | --- | --- | --- | --- |
|  |  |  |  |  |  |  | **Genotyping** |  | ***3DL1L1*** | ***3DL1S1*** | ***3DS1S1*** |  | ***3DL1L1*** | ***3DL1S1*** | ***3DS1S1*** |
| **Jennes** | **2006** | TanZania | African | 41 | 20 | 21 | PCR-SSP | 6 | 17 | 3 | 0 |  | 20 | 1 | 0 |
| **Boulet** | **2008** | Canada | Caucasian | 384 | 304 | 80 | PCR-SSP | 7 | 195 | 93 | 16 |  | 45 | 24 | 11 |
| **Guerin** | **2011** | Italy | Caucasian | 236 | 192 | 44 | PCR-SSP | 8 | 123 | 56 | 13 |  | 24 | 11 | 9 |
| **Habegger** | **2013** | Argentina | Caucasian | 123 | 100 | 23 | PCR-SSP | 7 | 55 | 42 | 3 |  | 3 | 17 | 3 |
| **Chavan** | **2014** | India | Asian | 94 | 47 | 47 | PCR-SSP | 7 | 13 | 24 | 10 |  | 5 | 35 | 7 |
| **Tallon** | **2014** | Canada | Caucasian | 168 | 74 | 94 | PCR-SSP | 7 | 41 | 31 | 2 |  | 55 | 27 | 12 |
| **Naranbhai** | **2016** | South Africa | African | 309 | 154 | 155 | PCR-SSO | 8 | 141 | 12 | 0 |  | 145 | 8 | 0 |
| **Zwolinska** | **2016** | Poland | Caucasian | 577 | 459 | 118 | PCR-SSP | 6 | 287 | 150 | 22 |  | 81 | 34 | 3 |
| **Jackson** | **2017** | Canada | Caucasian | 545 | 439 | 106 | PCR-SSP | 8 | 279 | 138 | 22 |  | 60 | 34 | 12 |
| **Rallon** | **2017** | Spain | Caucasian | 90 | 61 | 29 | PCR- SSO | 5 | 30 | 25 | 6 |  | 18 | 10 | 1 |

N; number of samples; HIV: Human Immunodeficiency Virus; HIVI: HIV-Infected; HESN: *HIV-exposed seronegative;* PCR-SSP: Polymerase Chain Reaction –Sequence Specific Primer;

PCR-SSO: Polymerase Chain Reaction-Sequence Specific Oligonucleotides; CB: Clark-Baudouin.
