# Supplementary material for: Effects of the killer immunoglobulin–like receptor (KIR) polymorphisms on HIV acquisition: A meta-analysis
Source: PLoS One. 2019 Dec 2;14(12):e0225151. doi: 10.1371/journal.pone.0225151 (PMC6886768; doi:10.1371/journal.pone.0225151)
Supplement: S5 Table — (DOCX) [file pone.0225151.s006.docx]

**Supplementary Table**

**S5 Table** Tests of interaction

| **KIR polymorphisms** |  |  |  |  |  |  | Uncorrected | Corrected |
| --- | --- | --- | --- | --- | --- | --- | --- | --- |
|  | a |  | b | OR^a^ | v | OR^b^ | P_interaction_ | P_interaction BC_ |
| *KIR* *gene content* |  |  |  |  |  |  |  |  |
| *2DL2* | Caucasian | v | Asian | 1.36 | v | 0.88 | 0.12 | > 1 |
| *2DL3* | Caucasian | v | African | 0.19 | v | 1.23 | **10^-5^** | **10^-4^** |
| *2DS1* | Caucasian | v | Asian | 1.71 | v | 0.90 | 0.29 | > 1 |
| *2DS1* | Caucasian | v | African | 1.71 | v | 1.04 | 0.10 | 1 |
| *KIR3DL1/S1 genotype* |  |  |  |  |  |  |  |  |
| *3DL1L1* | Asian | v | Caucasian | 3.21 | v | 1.20 | 0.10 | 1 |
| *3DL1L1* | Asian | v | African | 3.21 | v | 0.67 | 0.12 | > 1 |
| *3DL1S1* | Asian | v | Caucasian | 0.36 | v | 1.07 | **0.02** | 0.2 |
| *3DL1S1* | African | v | Asian | 1.73 | v | 0.36 | **0.012** | 0.12 |
| *3DS1S1* | Asian | v | Caucasian | 1.54 | v | 0.45 | 0.069 | 0.69 |
| *3DS1S1* | Asian | v | Caucasian* | 1.54 | v | 0.37 | **0.014** | 0.14 |

v: versus; OR: odds ratio; a: subgroup with significant ORs; b: subgroup with non-significant ORs; P-values were Bonferroni-corrected (_BC_).

Values in bold indicate significance (P < 0.05) of associations (under OR^a^) and P-values (corrected and uncorrected); *: post outlier.
