# Supplementary material for: Effects of the killer immunoglobulin–like receptor (KIR) polymorphisms on HIV acquisition: A meta-analysis
Source: PLoS One. 2019 Dec 2;14(12):e0225151. doi: 10.1371/journal.pone.0225151 (PMC6886768; doi:10.1371/journal.pone.0225151)
Supplement: S6 Table — (DOCX) [file pone.0225151.s007.docx]

**Supplementary Table**

**S6 Table** Comparison of two meta-analyses based on methodology

| **Parameter** | **This study** | **HCV clearance** |
| --- | --- | --- |
| Databases | PubMed, Science Direct and  Google Scholar | PubMed, EMBASE and ISI Web of Knowledge |
| Number of included studies | 13 | 16 |
| Number of *KIR* genes | 13 | 15 |
| *KIR3DL1/S1* genotype | yes | no |
| Bonferroni correction | yes | no |
| Test of interaction | yes | no |
| Outlier treatment | yes | no |
| I^2^ | yes | yes |
| Mantel-Haenszel and  DerSimonian-Laird | yes | yes |
| CB | yes | no |

I^2^; measure of variability; CB: Clark-Baudouin; EMBASE: Excerpta Medica dataBASE; ISI: Institute of Scientific Information
