# Supplementary material for: Effects of the killer immunoglobulin–like receptor (KIR) polymorphisms on HIV acquisition: A meta-analysis
Source: PLoS One. 2019 Dec 2;14(12):e0225151. doi: 10.1371/journal.pone.0225151 (PMC6886768; doi:10.1371/journal.pone.0225151)
Supplement: Attachment — Submitted filename: Responses to editorial comments 26 June 2019.docx [file pone.0225151.s008.docx]

Responses to editorial comments

26 June 2019

Title:

Effects of the killer immunoglobulin–like receptor (*KIR*) polymorphisms on HIV acquisition: a meta-analysis

**Editorial comments**

You currently have two versions of your manuscript included in your submission. Please ensure that only one copy of the manuscript is included with your submission files.

**Responses**

We apologize for the two versions. Here, we include only the amended version as of 26 June 2019. Thus, this re-submission has only one copy of the manuscript.

**Editorial comments**

To comply with the items on the PRISMA checklist, please provide the following information in your manuscript: a description of who assessed studies for inclusion, the results of the quality analysis for each individual study, and the list of excluded articles (the S1 List file is missing).

**Responses**

We supplied the information as to who assessed the studies for inclusion, who performed the quality analysis.

We also apologize for not including the S1 List; it is now included in this re-submission.

INCLUSION

LINES 159-160

SC and NP independently decided on which articles were to be included. This was then discussed in order to reach an agreement; otherwise, NS adjudicated so that consensus was obtained.

QUALITY ANALYSIS

LINE 179

SC and NP assessed the methodological quality of the included studies.
