# Supplementary material for: Effects of the killer immunoglobulin–like receptor (KIR) polymorphisms on HIV acquisition: A meta-analysis
Source: PLoS One. 2019 Dec 2;14(12):e0225151. doi: 10.1371/journal.pone.0225151 (PMC6886768; doi:10.1371/journal.pone.0225151)
Supplement: Attachment — Submitted filename: Responses to reviewer_31_AUG_2019.docx [file pone.0225151.s009.docx]

Responses to reviewer’s comments

PONE-D-19-15826
**Effects of the killer immunoglobulin–like receptor (*KIR*) polymorphisms on HIV acquisition: a meta-analysis**

Note: Texts from the line number below refer to non-track version of the manuscript.

**Reviewer #1**: The authors have done a terrific job at reviewing the relevant literature on the role of KIR polymorphisms in HIV acquisition, and, more importantly, at extracting the data and meta-data from these studies to perform a meta-analysis. The meta-analysis was satisfactorily performed. Scoring and ranking studies according to the CB scale lends credence to the meta-analysis to the extent that it establishes the quality of the "input" studies.

A major finding of the study is that the 2DL3 polymorphism is significantly protective against HIV acquisition even after Bonferroni correction. This was established only after outlier removal where outlier studies were identified by heterogeneity analysis and removed accordingly. The subsequent post-outlier treatment analysis yielded a strong inference that 2DL3 is indeed protective against HIV acquisition.

There are two very minor suggestions that may strengthen the paper a little bit:

**COMMENT:**

1) Clarification and interpretation of the significant interaction result between Caucasians and Africans. What does this mean even simply at an epidemiological level?

RESPONSE:

We clarify and interpret the significant interaction result between Caucasians and Africans. Prevalence of HIV in Africa is higher compared to that in North America and Europe (Caucasians) [1]. This epidemiological finding suggests less risk for Caucasians and higher risk for Africans which agrees with our 2DL3 result that protects Caucasians (OR, 0.19) from risk of HIV acquisition.

LINES 306-308

Extent of the significant Caucasian effect is thus placed in context when compared with its non-significant African counterpart.

**COMMENT:**
2) The Benjamini-Hochberg false discovery rate (BH FDR) is often a desirable alternative to the more rigorous Bonferroni correction in that it represents a nice balance between guarding against false discoveries and making true discoveries, as opposed to simply guarding against false discoveries in a relatively more conservative manner (as in the Bonferroni correction). This is not necessary for the authors to implement but its implementation may yield more results surviving multiple correction if the BH FDR is used.

RESPONSE:

We sincerely thank the reviewer for the insight provided about correcting for multiple comparisons. We did implement BH-FDR and obtained 11 significant P-values against the two with the Bonferroni correction. Then we found that explaining the 11 significant outcomes did not readily converge with the epidemiology and physiology literature. From the BH-FDR approach, this made the explanations messy which muddled the principal message of our study. Thus, we found that applying the Bonferroni correction, conservative as it is, readily lent more credence to our main message with better support from the literature.

*The Table at the end of this document puts the BH-FDR results in column

**Reviewer #2:** This is an interesting meta-analysis for effects of genetic variants on HIV acquisition.
The paper was well written and methods were rigorously used and described.

**COMMENT:**

A minor comment regarding the Galbraith plot. Was it based on an specific protective size of effect?

RESPONSE:

We thank the reviewer for this question. The Galbraith plot is mainly used to find source(s) of heterogeneity in meta-analysis. Thus, it would identify which component study/studies are outliers. In calculating the values along the Y-axis (log OR / SE), we indeed use the range of effect sizes (which maybe from protective to increased risk) from all the included studies in order to derive the log values. Rather than the specific protective size of effect as basis, it is the consequence of outlier analysis, which the Galbraith plot is the main instrument in identifying the outlier(s).

**COMMENT:**

Please define in the text if you did so. It is advisable to the authors include the size of effect plot (arc) on Figure 3.

RESPONSE:

The reason we use the log OR /SE is to derive positive and negative confidence limits for the Galbraith plot along the y-axis. Unfortunately, size of effect plot (arc) did not allow the above when we attempted to do so.

**COMMENT:**

Define Zwoliska in the same figure legend. This unique point was responsible for the 45% heterogeneity between studies.

RESPONSE: In the legend of figure 3 (below) we define the role of the outlying study, Zwolinska et al in terms of the heterogeneity in the Caucasian forest plot of the 3DS1S1 genotype comparison.

LINES 706-710

Figure 3: Galbraith plot analysis to detect the source of heterogeneity among Caucasian studies; the study above the +2 confidence limit is the outlier, Zwolinska et al [44]; whose presence in the PRO forest plot (Figure 2) accounts for 50% of the heterogeneity. Removal of this study [44] from the PSO forest plot (Figure 4) reduced the heterogeneity to 5%. OR: odds ratio; SE: standard error.

**COMMENT:**

There was not mention on the discussion regarding the advantage or disadvantages of using sensitivity approach versus eliminating the outlier.

RESPONSE: We thank the reviewer for this comment. We explain the pros and cons of using sensitivity analysis in the Discussion. Mainly, pooled ORs need to be tested for their stability. We address the question: will any study-specific OR be responsible for the instability of the pooled OR? This is tested simply by omitting one study at a time then recalculating the pooled OR without that study.

LINES 356-363

The advantage or disadvantages of using sensitivity approach versus eliminating the outlier is contextualized in terms of the following: Sensitivity treatment evaluates robustness of the pooled ORs while outlier elimination addresses heterogeneity. Favorable outcome of sensitivity analysis is robustness, where no study contributed to instability of the results. On the other hand, favorable outcomes of outlier treatment involve both heterogeneity and significance. In our study, heterogeneity was either reduced or eliminated; significance was intensified. These effects from outlier treatment and those from sensitivity analysis, contribute to strengthening the evidence that we present.

**COMMENT:**

Table 4 should clarify if the numbers in brackets are those affecting the results? (i.e.: Modifying the ORs in extreme results?). Can you clarify their significance?

RESPONSE: The bracketed numbers in the sensitivity analysis Table 4 are simply indications of the references that rendered non-robustness to the comparisons. Removing a study that results in a change of the pooled OR from significant (P < 0.05) to non-significant (P > 0.05) indicates non-robustness (instability) of the comparisons. The significance of the number brackets (Table 4) is a measure of stability/instability of the pooled results.

LINES 336-337

PRO: pre–outlier; PSO: post-outlier; the value in brackets indicate the references articles that contributed to instability of associations.

References

1. Maartens G, Celum C, Lewin SR (2014) HIV infection: epidemiology, pathogenesis, treatment, and prevention. Lancet 384: 258-271.

Attachment:
